# Supplementary material for: A Murine Model of High Dietary Histamine Intake: Impact on Histamine Contents and Release in Neural and Extraneural Tissues
Source: Nutrients. 2025 May 29;17(11):1851. doi: 10.3390/nu17111851 (PMC12156955; doi:10.3390/nu17111851)
Supplement: Supplementary file 1 [file nutrients-17-01851-s001.zip › nutrients-3645364-supplementary.pdf]

Supplementary Table S1: Energy content and composition of diets

The data were translated from the data sheets provided by Altromin GmbH (Lage, Germany) and Ssniff GmbH (Soest, Germany).

Altromin C 1072

**Convertible energy**	**kcal/kg** (given)	**kJ/kg** (calculated)	%
Carbon hydrates	2472	10343	66
Proteins	695	2908	18
Fat	593	2481	16
**All**	**3760**	**15732**	

**Amino acids**	**mg/kg**
Alanine	9121
Arginine	11114
Aspartic acid	15950
Cystine	2705
Glutamine	39075
Glycine	7092
Histidine	**4675**
Isoleucine	8762
Leucine	10791
Lysine	7763
Methionine	6370
Phenylalanine	6998
Proline	6174
Serine	9262
Threonine	7792
Tryptophan	2705
Tyrosine	5651
Valine	6028

**Fatty acids**	**mg/kg**
Arachidonic acid (C-20:0)	456
Eicosenoic acid (C-20:1)	570
Alpha-linoleic acid (C-18:3)	570
Linoleic acid (C-18:2)	29938
Palmitic acid (C-16:0)	7698
Stearic acid (C-18:0)	1711
Oleic acid (C-18:1)	13401

**Minerals**	**mg/kg**
Calcium	9548.0
Chlorine	3630.0
Magnesium	846.0
Phosphorus	7754.0
Potassium	7147.0
Sodium	4394.0
Sulphur	2241.74

**Trace elements**	**mg/kg**
Aluminium	5.81
Cobalt	0.13
Copper	12.52
Fluorine	4.17
Iodine	0.45
Iron	227.35
Manganese	103.99
Molybdenum	0.20
Selenium	0.25
Zinc	44.40

**Vitamins**	**mg/kg**
Biotin	0.3
Cholin chloride	1060
Folic acid	11
Nicotinic acid	75
Pantothenic acid	54
Vitamin A	15000 IU/kg
Vitamin B1	27
Vitamin B2	23
Vitamin B6	17
Vitamin B12	0.03
Vitamin C	21
Vitamin D3	500 IU/kg
Vitamin E	156
Vitamin K3	5

Ssniff V1124-000

Convertible energy	kJ/kg	%
Carbon hydrates	8680	62
Proteins	3640	26
Fat	1680	12
**All**	**14000**	

Amino acids	%
Alanine	1.02
Arginine	1.41
Aspartic acid	2.25
Cystine	0.39
Glutamine	4.65
Glycine	1.0
Histidine	***0.58***
Isoleucine	0.97
Leucine	1.69
Lysine	1.35
Methionine	0.48
Methionine+Cysteine	0.87
Phenylalanine	1.06
Proline	1.43
Serine	1.18
Threonine	0.85
Tryptophan	0.29
Phenylalanine+Tyrosine	1.81
Valine	1.08

Fatty acids	%
C 12:0	--
C 14:0	0.01
C 16:0	0.54
C 18:0	0.15
C 20:0	0.02
C 16:1	0.03
C 18:1	1.02
C 18:2	2.41
C 18:3	0.28

Minerals	%
Calcium	1.0
Calcium/Phosphorus	1.43:1
Magnesium	0.21
Phosphorus	0.7
Potassium	0.98
Sodium	0.24

Trace elements	mg/kg
Copper	15
Iodine	2.1
Iron	192
Manganese	62
Selenium	0.3
Zinc	89

Vitamins	mg/kg
Biotin	0.5
Cholin	1.4
Folic acid	7
Nicotinic acid	115
Pantothenic acid	40
Vitamin A	15000 IU/kg
Vitamin B1 (Thiamine)	18
Vitamin B2 (Riboflavine)	22
Vitamin B6 (Pyridoxine)	21
Vitamin B12 (Cobalamine)	0.01
Vitamin D3	1100 IU/kg
Vitamin E	110
Vitamin K	7
